# Spindle Cell Rhabdomyosarcoma of the Hand in a Full-Term Newborn: A Case Report

**DOI:** 10.7759/cureus.79670

**Published:** 2025-02-26

**Authors:** Meryem Khallouki, Maryem Aboudourib, Layla Bendaoud, Said Amal, Ouafa Hocar

**Affiliations:** 1 Department of Dermatology and Venereology, Mohammed VI University Hospital Center, Marrakesh, MAR

**Keywords:** chemotherapy, congenital rhabdomyosarcoma, diagnosis & prognosis, rhabdomyosarcoma, spindle cell rhabdomyosarcoma

## Abstract

Rhabdomyosarcoma (RMS) is a rare soft tissue tumor with the most common sites of origin in the genitourinary tract, head, and neck regions, and extremities are less commonly involved. Spindle cell RMS is a rare variant of RMS in infants. RMS is uncommon in the hand. We present a case of spindle cell RMS in the hand of a full-term female newborn with regional lymph node involvement. Based on histopathology, the diagnosis of spindle cell RMS was made in stage IRS 3 of the Intergroup Rhabdomyosarcoma Study Group (IRSG) staging system. The infant underwent complete surgical resection and axillary lymph node removal, followed by chemotherapy. She died from complications of chemotherapy a year after initiation of treatment. The age, gender, poor outcome, and site are unusual features for this type of RMS.

## Introduction

Congenital or neonatal rhabdomyosarcoma (RMS) is a rare soft tissue tumor with the most common sites of origin in the genitourinary tract, head, and neck regions, and extremities are less commonly involved. RMS is classiﬁed into three types, namely, embryonal, alveolar, and pleomorphic, as well as several subtypes and possible minor categories. Embryonal type variants and related tumors include botryoid, pleomorphic, clear cell, rhabdoid, spindle cell, and sclerosing types [1]. Congenital RMS represented 0.4% of all RMS cases in the Intergroup Rhabdomyosarcoma Study Group (IRSG) staging system. It primarily involves the head and neck region but may also occur at other sites such as the genitourinary tract, extremities, and trunk [2]. We present a case of spindle cell RMS in the hand of a full-term newborn female with regional lymph node involvement; the age, gender, and site are unusual features for this type of RMS.

This article was previously presented as a meeting abstract at the European Academy of Dermatology and Venereology (EADV) congress in Berlin in September 2023.

## Case presentation

**Figure 1:**
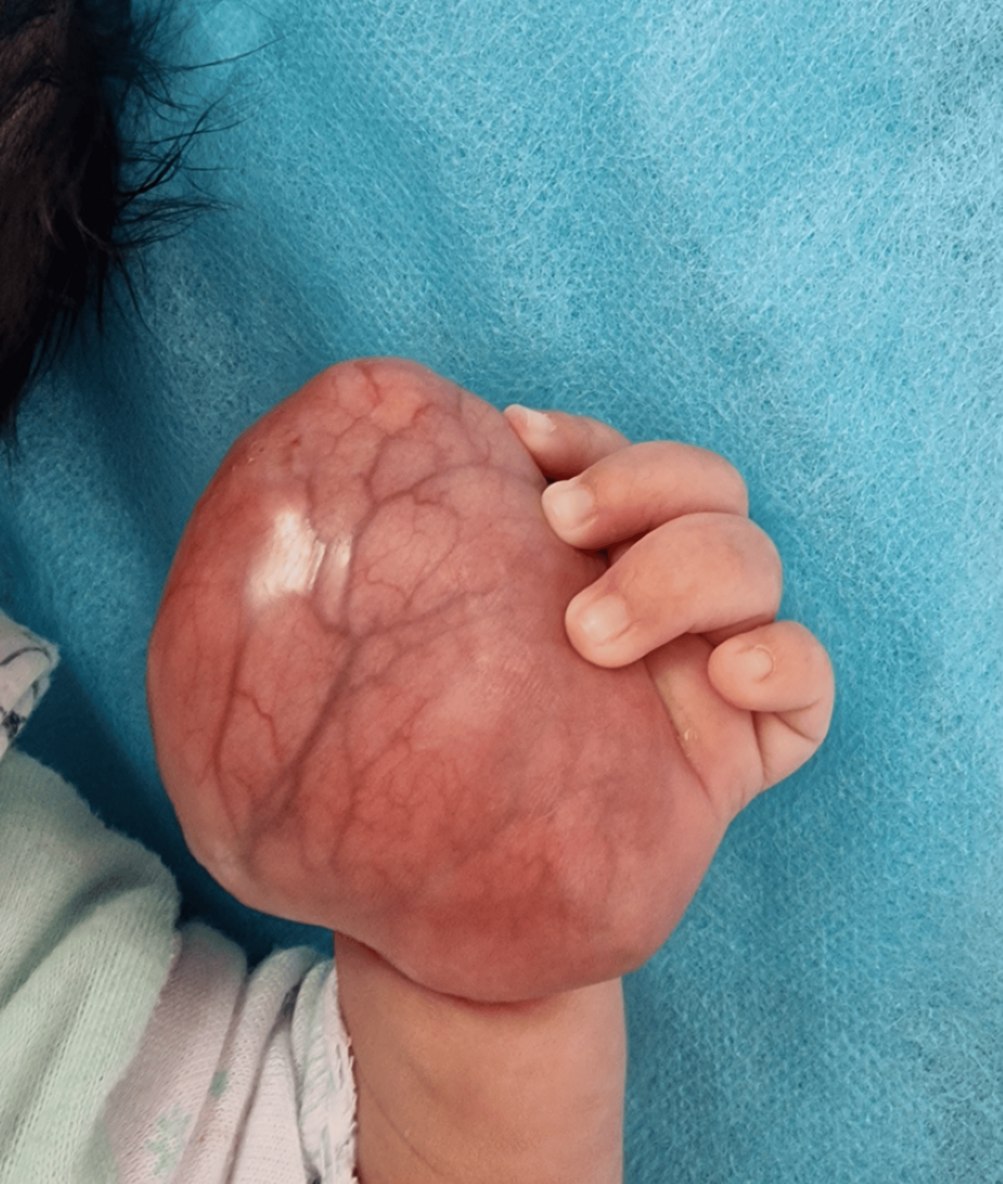
Clinical aspect of rhabdomyosarcoma of the left hand in a full-term newborn.

**Figure 2:**
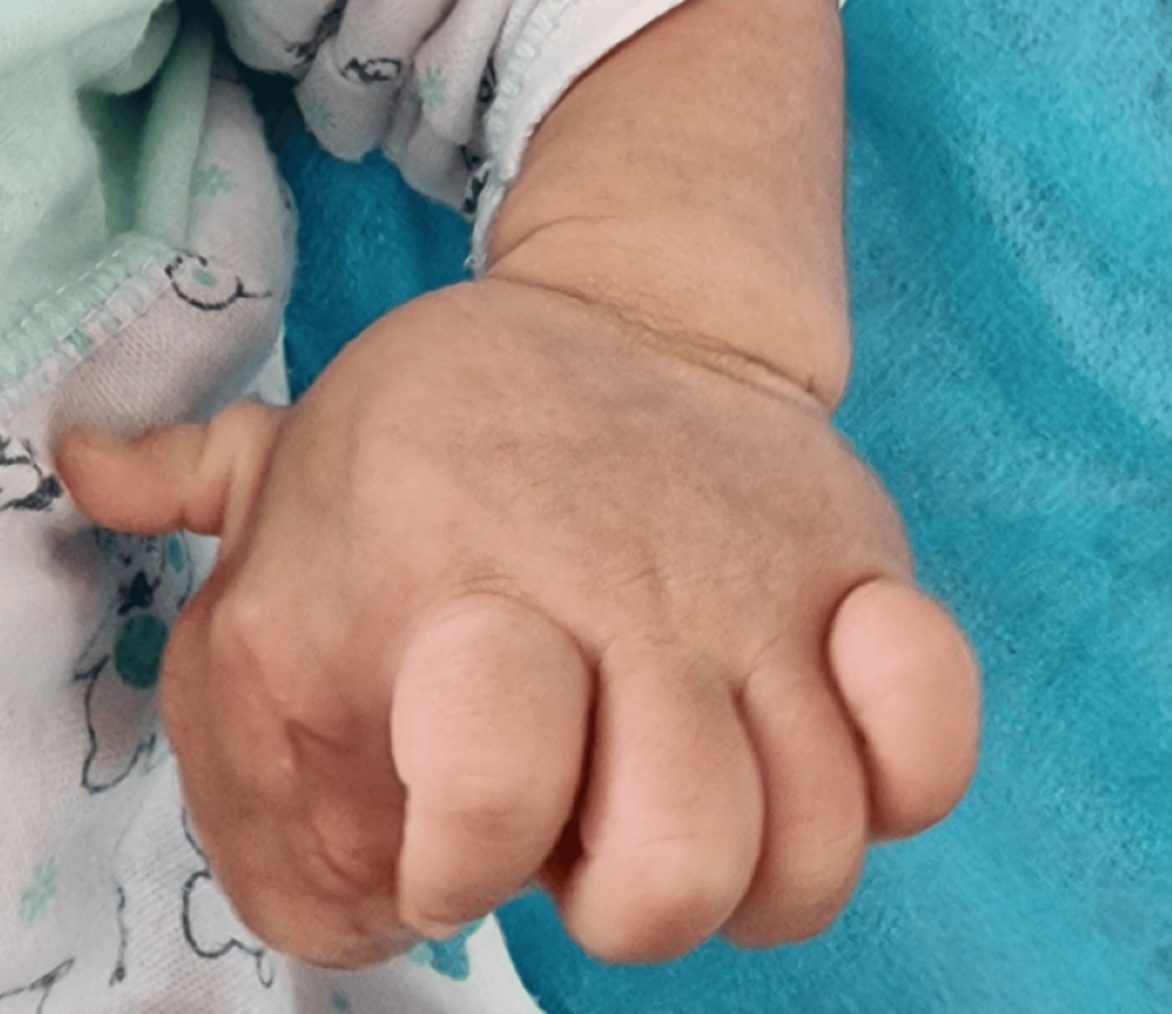
Clinical aspect of rhabdomyosarcoma of the left hand in a full-term newborn.

A five-day-old girl, born of non-consanguineous marriage, presented with swelling of the left hand since birth involving the entire palmar aspect of the hand distal to the wrist crease. Her mother’s full-term pregnancy was uncomplicated. On examination, the mass extended into the palmar aspect of the hand, measuring about 6 × 5 cm, firm, compressible, non-pulsatile, and non-transilluminant without any distal neurovascular deficit (Figure 1, Figure 2).

**Figure 3:**
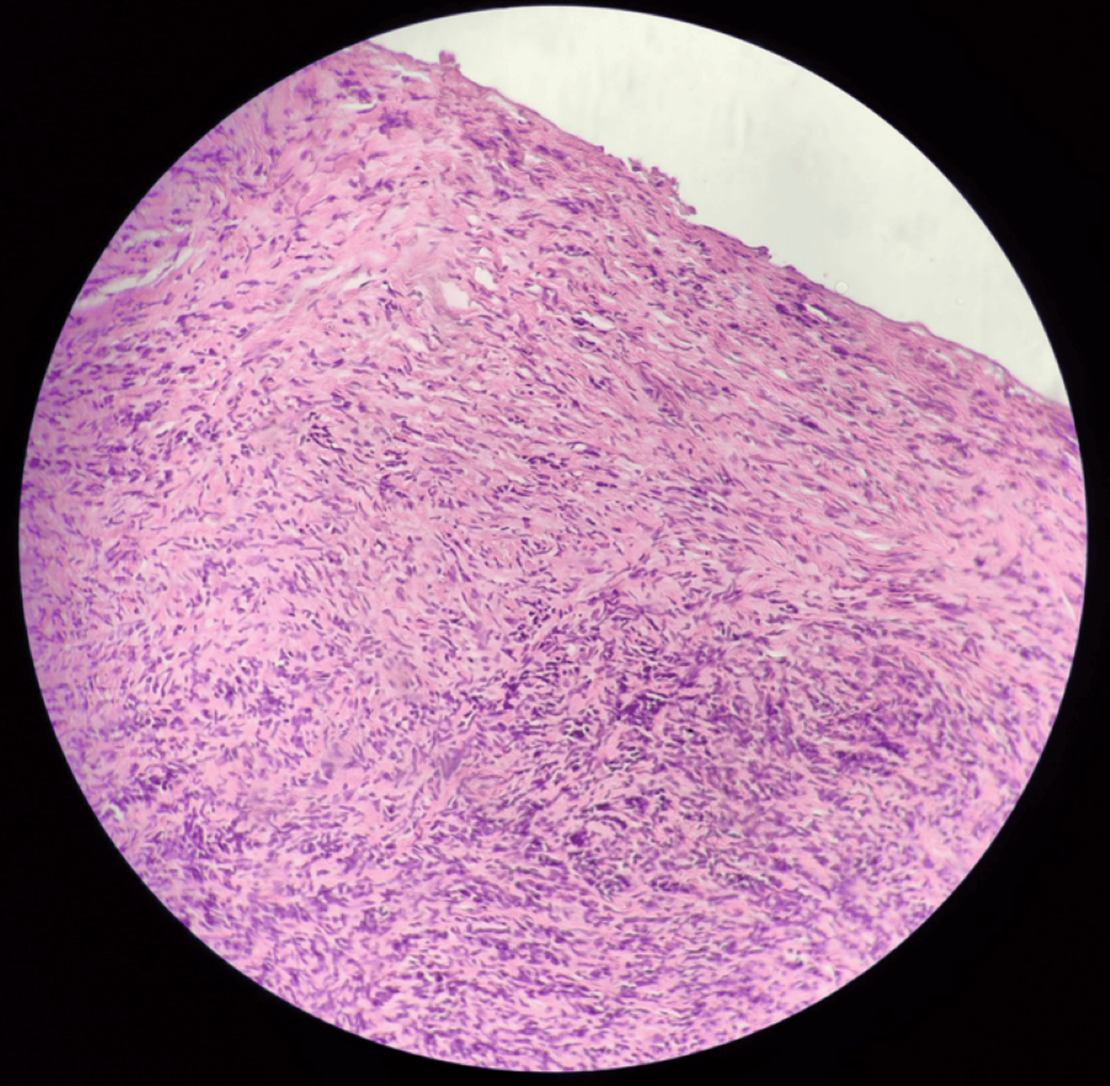
Microscopic examination of the hand lesion biopsy showing spindle cell tumor proliferation.

**Figure 4:**
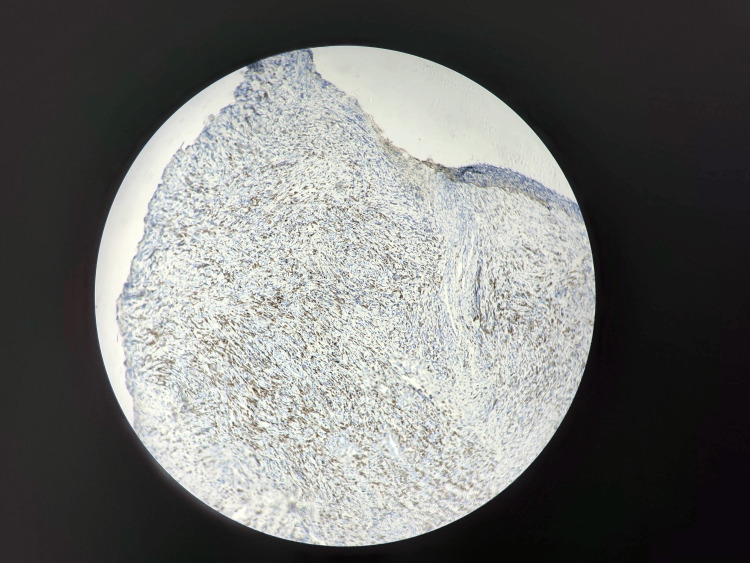
Immunohistochemical staining revealing positivity of anti-MYOD1.

There was palpable ipsilateral axillary lymphadenopathy. Routine neonatal laboratory values were normal: hemoglobin of 15 g/dL, platelets of 180 × 103/mcL, and fibrinogen of 2.45 g/L. Radiographs showed a soft tissue mass arising from the palmar aspect of the left hand without any bone involvement. Computed tomography (CT) angiogram showed a tissue formation centered on the palmar face of the left hand arriving at the dorsal face of the hand with widening of the intermetacarpal spaces, measuring approximately 57 x 45 x 43 mm. It was spontaneously isodense and heterogeneously enhanced after injection of pyruvate dehydrogenase complex (PDC), supplied by arterioles emanating from the ulnar and radial arteries with venous drainage towards the corresponding veins and individualization of superficial venous dilation. It is associated with ipsilateral axillary lymphadenopathy. The infant underwent a biopsy of the swelling in the hand, and histopathologic studies revealed a malignant spindle cell tumor proliferation; immunohistochemical staining revealed strong positivity of anti-Desmin and anti-MYOD1 (Figure 3, Figure 4).

VGLL2/NCOA2 molecular research was desirable but not available in our center. Based on histopathology, the diagnosis of spindle cell RMS was made in stage IRS 3 of the IRSG staging system. The IRSG study established a staging and grouping system from evaluated treatment protocols for RMS to determine prognosis and guide therapy. It included clinical examination, radiologic assessment of the primary site and regional lymph nodes, and evaluation of surgical margins following upfront resection. The infant was referred for complete surgical resection and axillary lymph node removal, followed by chemotherapy. Histological analysis of the surgical specimen showed an infiltrating spindle cell tumor proliferation with lymph node localization of RMS. Immunohistochemical staining revealed the positivity of anti-Desmin and anti-Myod1. The infant underwent chemotherapy after surgery for a year and died from complications of the chemotherapy.

## Discussion

RMS is a malignant mesenchymal tumor with striated muscle differentiation. It is commoner in young children with a peak age of occurrence at four years old. Only 6% of all RMS cases occur in infancy. Males are affected more often, but there is no gender difference in recent reported neonatal cases [1]. RMS arising before the age of one month is termed congenital RMS. Congenital RMS represented 0.4% of all RMS cases in the IRSG. It primarily involves the head and neck region but may also occur at other sites, such as the genitourinary tract, extremities, and trunk [2]. Spindle cell RMS is a rare variant of RMS, accounting for up to 10% of cases in infants [3]. RMS is uncommon in the hand, and those affecting the extremities have been associated with poor prognosis as they appear to have a high risk of regional lymph node metastasis [4]. Superficial tumors may mimic an abscess or hemangioma with characteristics of rubor, tenderness, and an increase in local vascularity [5]. Clinically, its differential diagnoses are neuroblastoma, lymphoma, leukemia, neuroﬁbroma, hemangioma, and fibromatosis. The most common sites of metastatic involvement are soft tissues, serosal surfaces, lungs, bone marrow, and lymph nodes. In comparison with other soft tissue tumors, lymph node metastasis is frequent in RMS and is of great value in tumor staging [4].

Spindle cell RMS is a rare variant of RMS, accounting for up to 10% of cases in infants. In older children and adults, spindle cell RMS is associated with MYOD1 mutations and a poor prognosis. In infants, it is associated with recurring fusions involving NCOA2 and VGLL2. Reports in the literature suggest a favorable prognosis for this subset; however, little is known about the treatment and outcome data of infants with spindle cell RMS. These tumors are frequently found in axial locations and are almost always localized at presentation [6]. An international cohort of infants diagnosed with spindle cell RMS between 1997 and 2018 aged <12 months at the time of the diagnosis reported one patient with metastatic disease and 39 patients with localized disease, of which 10 patients had a location in the extremities. Only one infant had regional lymph node involvement (primary in the extremity). Patients were treated with a combination of therapies, including chemotherapy, surgical resection, and radiation therapy (RT) according to their IRS stage. Overall, 38/39 patients achieved complete response CR. Thirty-five patients had no relapse (92% of patients in CR), and 36 were alive in clinical remission at the last follow-up. Three patients died one of whom had N1 disease and died from metastatic relapse. The five-year event-free survival (EFS) was 86%, and the five-year overall survival (OS) was 91%, suggesting a favorable outcome for infants with spindle cell RMS, with lower rates of relapse and possibly death than combined histologic subtypes of infantile RMS. The extent of resection was defined as a prognostic factor. Based on this largest study of infants with spindle cell RMS, the common first-line treatment recommended is R0 (complete) resection (if feasible without mutilation) and systemic treatment with risk-adapted therapy using the VAC (vincristine, actinomycin, and cyclophosphamide) or IVA (vincristine, actinomycin, ifosfamide). Anthracyclines and external beam RT can be avoided in the majority of patients [6]. Our case is worthwhile to report because of the extreme rarity of RMS in neonates. Furthermore, to our knowledge, it is the second congenital RMS arising in an extremity with lymph node involvement. Poor prognosis factors in our case were the age ≤ 12 months and lymph node involvement. Most of the previously reported congenital RMS had male dominance, but our patient was female.

## Conclusions

Spindle cell and sclerosing RMS is a rare variant of RMS, which includes three distinct subtypes. It primarily involves the head and neck region but may also occur at other sites, such as extremities like a hand in neonates. Therefore, necessary diagnostic immunohistochemical staining should be done to investigate and manage better the disease. Infants with congenital spindle cell RMS present with localized disease, and the literature shows that infants with the localized type of RMS have favorable prognosis. However, little is known about the treatment and outcome data of infants with spindle cell RMS.

## References

[1] Esmaeili H, Azimpouran M (2017). Congenital embryonal rhabdomyosarcoma; multiple lesions. Int J Surg Case Rep.

[2] Tewari VV, Mehta R, Tewari K (2016). Congenital embryonal rhabdomyosarcoma presenting as a cutaneous nodule in a neonate. Int J Clin Pediatr.

[3] Slater O, Gains JE, Kelsey AM (2022). Localised rhabdomyosarcoma in infants (<12 months) and young children (12-36 months of age) treated on the EpSSG RMS 2005 study. Eur J Cancer.

[4] Terwisscha van Scheltinga CE, Spronk P, van Rosmalen J (2014). Diagnosis and treatment of lymph node metastases in pediatric rhabdomyosarcoma in the Netherlands: a retrospective analysis. J Pediatr Surg.

[5] Ognjanovic S, Linabery AM, Charbonneau B, Ross JA (2009). Trends in childhood rhabdomyosarcoma incidence and survival in the United States, 1975-2005. Cancer.

[6] Whittle S, Venkatramani R, Schönstein A (2022). Congenital spindle cell rhabdomyosarcoma: an international cooperative analysis. Eur J Cancer.

